# Subcutaneous Fat Area Can Be as a Predictors of Drainage Volume After Lobectomy for Lung Cancer

**DOI:** 10.1111/1759-7714.70114

**Published:** 2025-06-21

**Authors:** Jie Xu, Shuai Yuan, Xiaopeng An, Jie Dong

**Affiliations:** ^1^ Department of Lung Cancer, Tianjin Medical University Cancer Institute & Hospital, National Clinical Research Center for Cancer, Key Laboratory of Cancer Prevention and Therapy Tianjin's Clinical Research Center for Cancer Tianjin China; ^2^ Tianjin Medical University Cancer Institute & Hospital Tianjin Medical University Tianjin China; ^3^ Department of Nutrition Therapy, Tianjin Medical University Cancer Institute & Hospital, National Clinical Research Center for Cancer, Key Laboratory of Cancer Prevention and Therapy Tianjin's Clinical Research Center for Cancer Tianjin China

**Keywords:** drainage volume, length of stays, lobectomy, lung cancer, subcutaneous fat area

## Abstract

**Objective:**

Currently, no uniform standard exists for the maximum drainage volume permitting chest tube removal following lobectomy in lung cancer patients, and limited research has explored factors influencing postoperative drainage. This study aimed to investigate the relationship between subcutaneous fat area (SFA) and postoperative drainage volume.

**Methods:**

We conducted a retrospective analysis of 509 lung cancer patients who underwent video‐assisted thoracoscopic lobectomy. Clinical characteristics, postoperative outcomes (length of stay, hospitalization expenses), blood parameters, chest tube duration, 3‐day postoperative drainage volume, and SFA were recorded. Predictive factors for drainage volume were identified using univariate and multivariate logistic regression analyses. SFA was measured at the level of the 12th thoracic vertebra cross‐section using preoperative CT imaging.

**Results:**

The analysis revealed significant positive correlations between chest tube duration and both length of stay (*p* < 0.001) and hospitalization expenses (*p* < 0.001). Chest tube duration (< 3 vs. ≥ 3 days) was primarily determined by 3‐day postoperative drainage volume (574 ± 252 vs. 885 ± 362 mL; *p* < 0.001). Univariate analysis demonstrated that 3‐day postoperative drainage volume correlated with age (*p* < 0.001), gender (*p* = 0.002), pathological type (*p* < 0.001), diabetes (*p* = 0.026), hypertension (*p* = 0.011), and SFA (*p* < 0.001). Multivariate logistic regression confirmed that age ≥ 65 years (*p* = 0.016), small cell lung cancer (SCLC; *p* = 0.022), and SFA ≥ 100 cm^2^ (*p* = 0.005) were independently associated with postoperative drainage volume ≥ 650 mL.

**Conclusions:**

SFA significantly correlated with 3‐day postoperative drainage volume and may serve as a predictor for drainage volume following lobectomy in lung cancer patients. This association highlights its utility in guiding chest tube removal timing and potentially reducing the risk of pleural effusion recurrence.

## Graphical abstract

Subcutaneous fat area (SFA) significantly correlated with 3‐day postoperative drainage volume and may serve as a predictor for drainage volume following lobectomy in lung cancer patients. This association highlights its utility in guiding chest tube removal timing and potentially reducing the risk of pleural effusion recurrence; it provides clinical value for preoperative risk assessment and postoperative management.

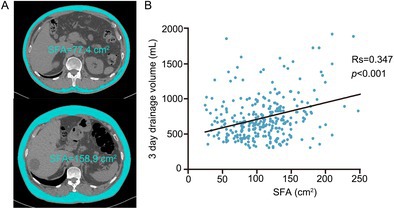

## 1 Introduction

Lung cancer ranks among the most prevalent malignant tumors globally, with non‐small cell lung cancer (NSCLC) comprising 85%–90% of cases and exhibiting high morbidity and mortality [1, 2]. Surgical resection remains the primary treatment for Stage I–IIIA disease [3]. Over the past two decades, video‐assisted thoracoscopic lobectomy (VATS) has evolved into a mature technique. As a minimally invasive approach, VATS eliminates rib spreading and utilizes smaller incisions (5–8 cm), thereby improving therapeutic efficacy, reducing complications, alleviating postoperative pain, accelerating recovery, and shortening hospital stays [4, 5]. Postoperative pleural effusion represents the most frequent complication following VATS lobectomy and a key determinant of prolonged hospitalization [6]. Consequently, chest tube drainage remains a standard postoperative practice [7]. Extended chest tube retention exacerbates patient discomfort and elevates complication risks. Evidence indicates that a single chest tube—compared to traditional dual‐tube placement—effectively evacuates pleural effusion while reducing postoperative pain and shortening hospital stays [7, 8].

Although no universal standard exists for pleural fluid output thresholds permitting chest tube removal (reported ranges: 200–500 mL/day, median: 400 mL/day) [9, 10], high‐volume postoperative pleural effusion remains a well‐established cause of delayed tube removal. While chest tube milking may prevent catheter occlusion, it can paradoxically increase drainage output; consequently, routine milking is not recommended [11]. Current research primarily focuses on chest tube management strategies—including removal timing and drainage system design—yet limited evidence exists regarding factors influencing pleural effusion volume itself. Obesity‐driven inflammatory states may heighten postoperative fluid production; although it is generally believed that subcutaneous fat is less harmful in terms of metabolism than visceral fat, a high amount of subcutaneous fat is also associated with a pro‐inflammatory state and may affect wound healing and tissue repair, motivating our investigation into subcutaneous fat area (SFA) as a quantifiable biomarker of drainage volume. Although L3‐level body composition analysis remains the reference standard for whole‐body assessment, preoperative staging CT in lung cancer routinely captures T12 but not L3 [12]. Robust validation studies support T12 as a reliable alternative for adiposity metrics when L3 is unavailable [13]. Thus, SFA was measured at T12 using axial CT images to leverage existing clinical data.

## 2 Patients and Methods

### 2.1 Patients

We retrospectively collected and analyzed the medical records of patients who underwent VATS for lung adenocarcinoma and small cell lung cancer (SCLC) at Tianjin Cancer Hospital between September 2022 and January 2024. A total of 509 patients were included in this study, and all patients were treatment naive. Pulmonary lobectomy and systematic nodal dissection were performed in all patients, and a drainage chest tube was routinely placed after surgery and excluded patients with chylothorax, hemorrhage, air leakage, chest tube milking, wound infection, pleural adhesion, and atelectasis, as these factors could seriously affect the volume of pleural effusion drainage. The following data were collected: age, sex, history, tumor location, pathological parameters, body mass index (BMI), percentage predicted forced expiratory volume in 1 s (FEV1.0%), chest tube days, length of stay, 3‐day drainage volume (mL), and blood parameters including levels of serum prealbumin (PALB), albumin (ALB), hemoglobin (HB), C‐reactive protein (CRP), total cholesterol (TC), triglyceride (TG), high‐density lipoprotein (HDL), and low‐density lipoprotein (LDL).

Meanwhile, to verify the results of the retrospective study, we prospectively collected information from 44 patients with adenocarcinoma. The inclusion criteria and exclusion criteria were consistent with those of the retrospective study, and the correlation between the 3‐day drainage volume and SFA was analyzed.

### 2.2 CT Image Analysis

We evaluated the SFA at the 12th thoracic vertebra (T12) level using diagnostic CT within 1 month of surgery. Slice‐O‐Matic software (V4.3; Tomovision, Montreal, QC, Canada) to evaluate T12 two consecutive images, and adipose tissue cross‐sectional areas were calculated using standard Hounsfield unit (HU) thresholds of −190 to −30 HU for SAT [14].

### 2.3 Statistical Analysis

All data in this study were evaluated using the SPSS Version 23.0 software (IBM SPSS Inc., Chicago). Descriptive statistics are presented as mean ± standard deviation for continuous variables and percentages for categorical variables. Continuous variables were analyzed by analysis of variance or student *t*‐test, and categorical variables were analyzed by *χ* ^2^ test for contingency table data. The best cut‐off point values for SFA were determined by receiver operating characteristic (ROC) analysis. The correlation between 3‐day postoperative drainage volume and SFA was assessed using Spearman's rank correlation coefficient. Univariate analysis was performed to identify the predictive factors of postoperative drainage volume, and multivariate analysis was performed using logistic regression analysis with hazard ratios and 95% confidence intervals (CI). A level of *p* < 0.05 was considered statistically significant.

## 3 Results

### 3.1 Chest Tube Days Extend Length of Stay and Increase Hospitalization Expenses

The average postoperative chest tube days in 509 patients was 3 days. Therefore, we analyzed the relationship between the clinical characteristics of the patients and chest tube days (Table 1). The results showed that the chest tube days were significantly correlated with gender (*p* = 0.001) and SCLC (*p* = 0.004). At the same time, the chest tube days ≥ 3 were highly correlated with the postoperative 3‐day drainage volume (574 ± 252 vs. 885 ± 362 mL, *p* < 0.001), and significantly increased the length of stay (4.0 ± 0.7 vs. 6.5 ± 2.4, *p* < 0.001) and hospitalization expenses (69 262 ± 36 039 vs. 74 212 ± 10 157, RMB, *p* = 0.017).

**TABLE 1:** Correlation between patient characteristics and chest tube days.

	Chest tube days		*p*
	< 3 (*n* = 370)	≥ 3 (*n* = 139)	
Age (years)			0.077
< 65	253 (68.4)	85 (61.2)	
≥ 65	117 (31.6)	54 (38.8)	
Gender			0.001**
Male	121 (32.7)	67 (48.2)	
Female	249 (67.3)	72 (51.8)	
Pathological parameters			0.004**
Adenocarcinoma	323 (87.3)	107 (77.0)	
SCLC	47 (12.7)	32 (23.0)	
Smoking history			0.243
No	243 (65.7)	86 (61.9)	
Yes	127 (34.3)	53 (38.1)	
Drinking history			0.068
No	301 (81.4)	104 (74.8)	
Yes	69 (18.6)	35 (25.2)	
Diabetes			0.085
No	316 (85.4)	111 (79.9)	
Yes	54 (14.6)	28 (20.1)	
Hypertension			0.225
No	244 (65.9)	86 (61.9)	
Yes	126 (34.1)	53 (38.1)	
EFV1%	88.0 ± 7.0	85.9 ± 9.4	0.423
Operative time (min)	132 ± 20	138 ± 21	0.566
3‐day drainage volume (mL)	574 ± 252	885 ± 362	< 0.001***
Length of stay	4.0 ± 0.7	6.5 ± 2.4	< 0.001***
Hospitalization expenses (RMB)	69 262 ± 36 039	74 212 ± 10 157	0.017*

Abbreviation: EFV1%, percentage predicted forced expiratory volume in 1 s.

*p < 0.05, **p < 0.01, and ***p < 0.001 (in bold).

### 3.2 Postoperative 3‐Day Drainage Volume Was Correlated With SFA

**FIGURE 1:**
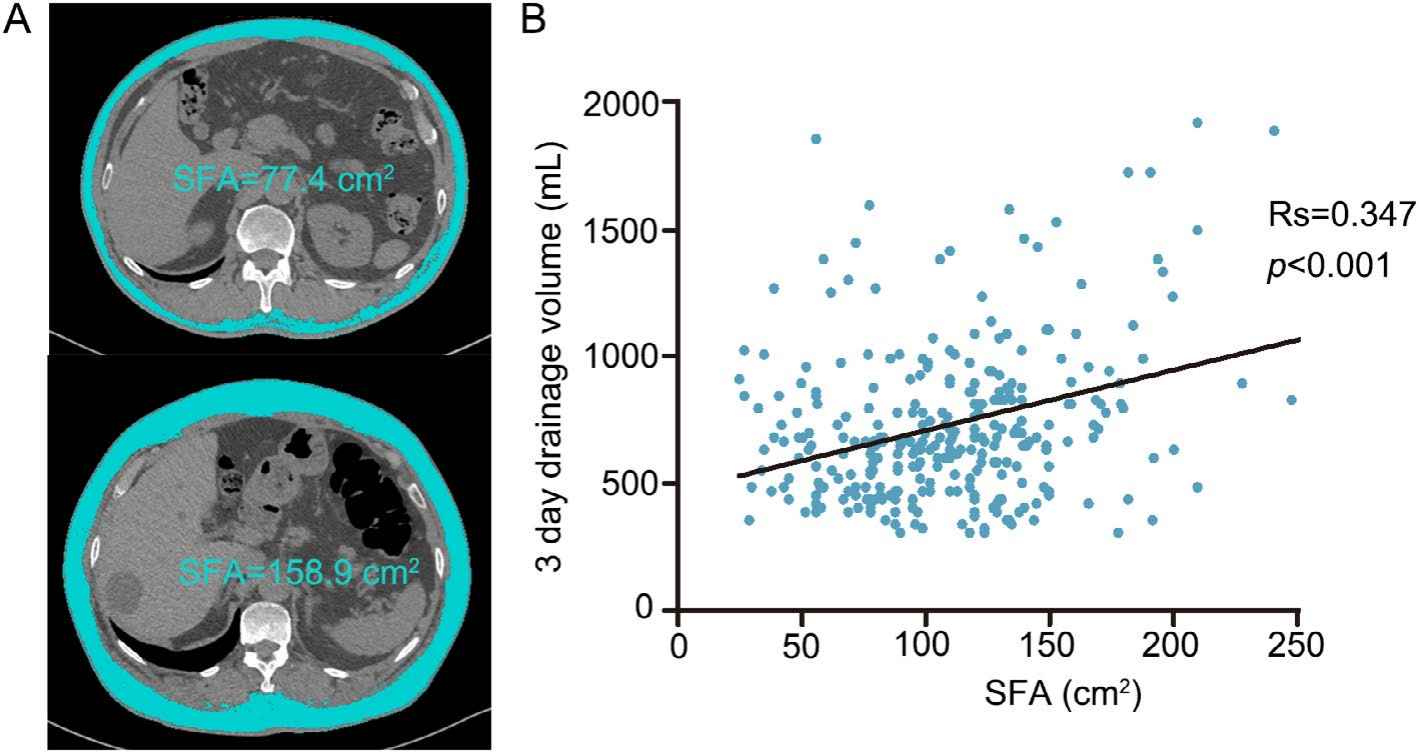
(A) Axial computed tomography images of the 12th thoracic vertebra region with subcutaneous fat area highlighted in blue; (B) Scatter plot highlights the relationship between drainage volume and SFA(Rs = 0.347, *p* < 0.001).

The mean value of 3‐day drainage volume was 659 ± 318 mL, and the median was 650 mL. Therefore, we divided 3‐day drainage volume into < 650 and ≥ 650 mL groups, and we compared the differences in clinical parameters between the two groups (Table 2). The analysis results showed that the levels of CRP (*p* = 0.011) in the patients with 3‐day drainage volume ≥ 650 mL group were significantly higher than those in the < 650 mL group. At the same time, we found that patients with 3‐day drainage volume ≥ 650 mL had a significantly higher value of SFA than patients with 3‐day drainage volume < 650 mL(109.1 ± 48.0 vs. 87.9 ± 35.7 cm^2^, *p* = 0.003), and as is shown in Figure 1, there was a positive correlation between them (*R*_s_ = 0.347, *p* < 0.001).

**TABLE 2:** Differences in clinical parameters between the 3‐day drainage volume < 650 and ≥ 650 mL.

	3‐day drainage volume (mL)		*p*
	< 650 (*n* = 264)	≥ 650 (*n* = 245)	
PALB (g/L)	0.25 ± 0.05	0.25 ± 0.05	0.526
ALB (g/L)	44.7 ± 3.6	44.6 ± 3.4	0.839
HB (g/L)	140.9 ± 76.8	138.3 ± 13.8	0.254
CRP (g/L)	1.7 ± 3.8	3.1 ± 13.1	0.011*
TC (mmol/L)	4.9 ± 1.1	5.0 ± 1.2	0.195
TG (mmol/L)	1.5 ± 1.4	1.6 ± 0.8	0.575
HDL (mmol/L)	1.4 ± 0.4	1.3 ± 0.4	0.773
LDL (mmol/L)	3.1 ± 0.9	3.2 ± 1.0	0.190
D‐dimer (mmol/L)	365 ± 403	417 ± 628	0.359
BMI (kg/m^2^)	24.2 ± 3.3	24.5 ± 3.6	0.132
SFA (cm^2^)	87.9 ± 35.7	109.1 ± 48.0	0.003**

Abbreviations: ALB, albumin; BMI, body mass index; CRP, C‐reactive protein; HB, hemoglobin; HDL, high‐density lipoprotein; LDL, low‐density lipoprotein; PALB, prealbumin; SFA, subcutaneous fat area; TC, total cholesterol; TG, triglyceride.

*p < 0.05, **p < 0.01, and ***p < 0.001 (in bold).

### 3.3 SFA Can Be Used as a Predictor of Postoperative 3‐Day Drainage Volume ≥ 650 mL

The best cut‐off value for SFA were determined at 100cm^2^ for 3‐day drainage volume ≥ 650 mL by using ROC analysis (*p* < 0.001, Figure S1A), and univariate and multivariate analyses were used to analyze the factors that may affect 3‐day drainage volume (Table 3). Univariate analysis showed that age ≥ 65 (*p* < 0.001), male (*p* = 0.002), SCLC (*p* < 0.001), diabetes (*p* = 0.026), hypertension (*p* = 0.011), and SFA ≥ 100cm^2^ (*p* < 0.001) all increased postoperative 3‐day drainage volume. However, multivariate analysis showed that in addition to age ≥ 65 (*p* = 0.016) and SCLC (*p* = 0.022), SFA ≥ 100 cm^2^ (*p* = 0.005) can be used as a predictor affecting 3‐day drainage volume.

**TABLE 3:** Univariate and multivariate analyses of 3‐day drainage volume ≥ 650 mL.

	Univariate	Multivariate	
	*p*	*p*	(95% confidence interval)
Age (< 65 vs. ≥ 65)	< 0.001***	0.016*	1.767 [1.181, 2.643]
Gender (male vs. female)	0.002**	0.219	0.760 [0.491, 1.177]
Pathological parameters (adenocarcinoma vs. SCLC)	< 0.001***	0.022*	2.334 [1.348, 4.042]
Smoking history (no vs. yes)	0.218		
Drinking history (no vs. yes)	0.119		
Diabetes (no vs. yes)	0.026*	0.970	1.001 [0.577, 1.772]
Hypertension (no vs. yes)	0.011*	0.138	1.382 [0.901, 2.120]
CRP (normal vs. abnormal)	0.194		
BMI (< 23.9 vs. ≥ 23.9)	0.196		
SFA (< 100 vs. ≥ 100 cm^2^)	< 0.001***	0.005**	1.243 [1.234, 3.286]

Abbreviations: BMI, body mass index; CRP, C‐reactive protein; SFA, subcutaneous fat area.

*p < 0.05, **p < 0.01, and ***p < 0.001 (in bold).

To further examine the correlation between SFA and 3‐day drainage volume, we prospectively collected samples from 44 cases of new lung cancer patients undergoing lobectomy and conducted correlation analysis. The analysis results were consistent with previous results, further confirming that SFA was significantly associated with postoperative drainage volume (*R*_s_ = 0.216, *p* = 0.002, Figure S1B).

In addition, PALB and HB decreased after operation in both groups (*p* < 0.001, Table S1), but PALB levels decreased more in patients with 3‐day drainage volume of ≥ 650 mL (*p* = 0.001). At the same time, the postoperative CRP and D‐dimer levels increased in both groups (*p* < 0.001), and the CRP levels increased more in patients with 3‐day drainage volume ≥ 650 mL (*p* < 0.006). This may mean that an increased drainage volume extends the chest tube days and increases the patient's inflammatory response, and in turn, increased inflammatory expression may also increase pleural fluid.

## 4 Discussion

Postoperative chest tube drainage remains a routine procedure following VATS lobectomy, facilitating evacuation of blood/air, preventing exudate reflux, maintaining intrathoracic negative pressure, promoting lung re‐expansion, and reducing infection risk [15]. However, prolonged drainage duration exacerbates postoperative pain, increases infection rates, extends hospitalization, and elevates costs—collectively impeding enhanced recovery [16]. With the continuous development of surgical technology and the concept of enhanced recovery after surgery (ERAS), the management of postoperative chest tubes has also improved, focusing on the size and model of chest tubes and the criteria for removal, but there are still controversies [17, 18]. In recent years, the general consensus among surgeons has been that one chest tube is superior to two, reducing pain and infection [19]. Regarding the type of chest tube, a 24Fr BD is currently considered safe and is generally used [20]. However, some studies have shown that the 19Fr BD has the advantage of effectively removing air and fluid [21], while other studies have shown that the 8F ultrafine chest tube can shorten the drainage time, reduce drainage flow, reduce pain, and is safe and effective [22]. Currently, there is no consensus on the criteria for chest tube extraction. Some studies suggest that less than 300 mL/d of chest drainage volume is appropriate because the daily filtration of pleural fluid is 350 mL physiologically, whereas others believe that 400–450 mL is safe [23, 24]. However, studies have shown that when the drainage volume is greater than 500 mL/d to remove the chest tube, some patients have a recurrence of the effusion, requiring further intervention [10]. Another important factor in removing a chest tube is the chemical signature of pleural fluid, and studies have shown that a ratio of pleural fluid to blood protein (PrRP/B) of less than 0.5 is a predictor of safety [25].

The removal of the chest tube was closely related to daily drainage volume, which was also confirmed in our study. Therefore, for patients with a high drainage volume, premature removal of the chest tube may lead to recurrent intervention. Few studies have analyzed the factors that cause excessive drainage volume. If the factors that cause excessive chest drainage can be identified, targeted attention can be paid to these patients to make it safer to remove the chest tube and avoid the recurrence of pleural fluid, whereas patients without risk factors can remove the chest tube as soon as possible, which may be one of the strategies for future individualized chest tube management. Obesity, accompanied by the accumulation of body fat, is an inflammatory state that can lead to an increase in inflammatory cytokines [26, 27], and high inflammatory state will increase the postoperative drainage volume. Therefore, we want to explore the relationship between the amount of subcutaneous fat and the postoperative drainage volume. The cross‐section of the third lumbar vertebra is the gold standard for evaluating the composition of the human body. When CT images of the third lumbar vertebra cannot be captured, the thoracic 12 level can be used as an alternative. Our findings suggest that age, gender, pathological classification, hypertension, and diabetes all affect the 3‐day drainage volume after surgery, but SFA can be a special predictor, it provides valuable information beyond BMI and traditional risk factors, emphasizing the importance of body composition analysis in surgical risk assessment. SFA seems to be particularly related to pleural space healing and fluid production. Perhaps SFA can be incorporated into clinical practice to improve the risk stratification and management of patients.

## 5 Conclusion

Postoperative chest drainage volume seriously affects the length of stay and hospitalization cost. Our study suggests that SFA can be a predictor of high flux pleural effusion, and early removal of the chest tube should be vigilant for these patients to prevent recurrence of pleural effusion and rehospitalization intervention.

## Author Contributions

**Jie Xu, Jie Dong:** conception and design, obtained funding and study supervision. **Shuai Yuan:** acquisition and interpretation of data. **Xiaopeng An:** data Analysis. **Jie Dong, Jie Xu:** drafting of the manuscript. **Jie Dong:** critical revision of the article for important intellectual content. Finally approved the final version of the manuscript.

## Ethics Statement

This study was conducted in accordance with the Declaration of Helsinki (revised in 2013). This study was retrospective and did not involve specimens and was approved by the ethics committee of Tianjin Medical University Cancer Institute and Hospital with exemption of informed consent.

## Conflicts of Interest

The authors declare no conflicts of interest.

## Supporting information


**Figure S1.** Reactions between GPTMS and mesh. (A) ROC curve of SFA for 3‐day drainage volume (*p* < 0.001). (B) Correlation of SFA in 44 cases of new lung cancer patients undergoing lobectomy to 3‐day drainage volume (*R*_s_ = 0.216, *p* = 0.002).

**Table S1.** Differences of pre‐ and postoperative in blood parameters in patients with 3‐day drainage volume < 650 and ≥ 650 mL. CRP, C‐reactive protein; HB, hemoglobin; ALB, albumin; PALB, prealbumin. *p < 0.05, **p < 0.01, and ***p < 0.001.

## Data Availability

The data underlying this article cannot be shared publicly due to the privacy of individuals that participated in the study, and our research is not yet complete.
