# Supplementary figures and images for: Subcutaneous Fat Area Can Be as a Predictors of Drainage Volume After Lobectomy for Lung Cancer
Source: Thorac Cancer. 2025 Jun 21;16(12):e70114. doi: 10.1111/1759-7714.70114 (PMC12181634; doi:10.1111/1759-7714.70114)

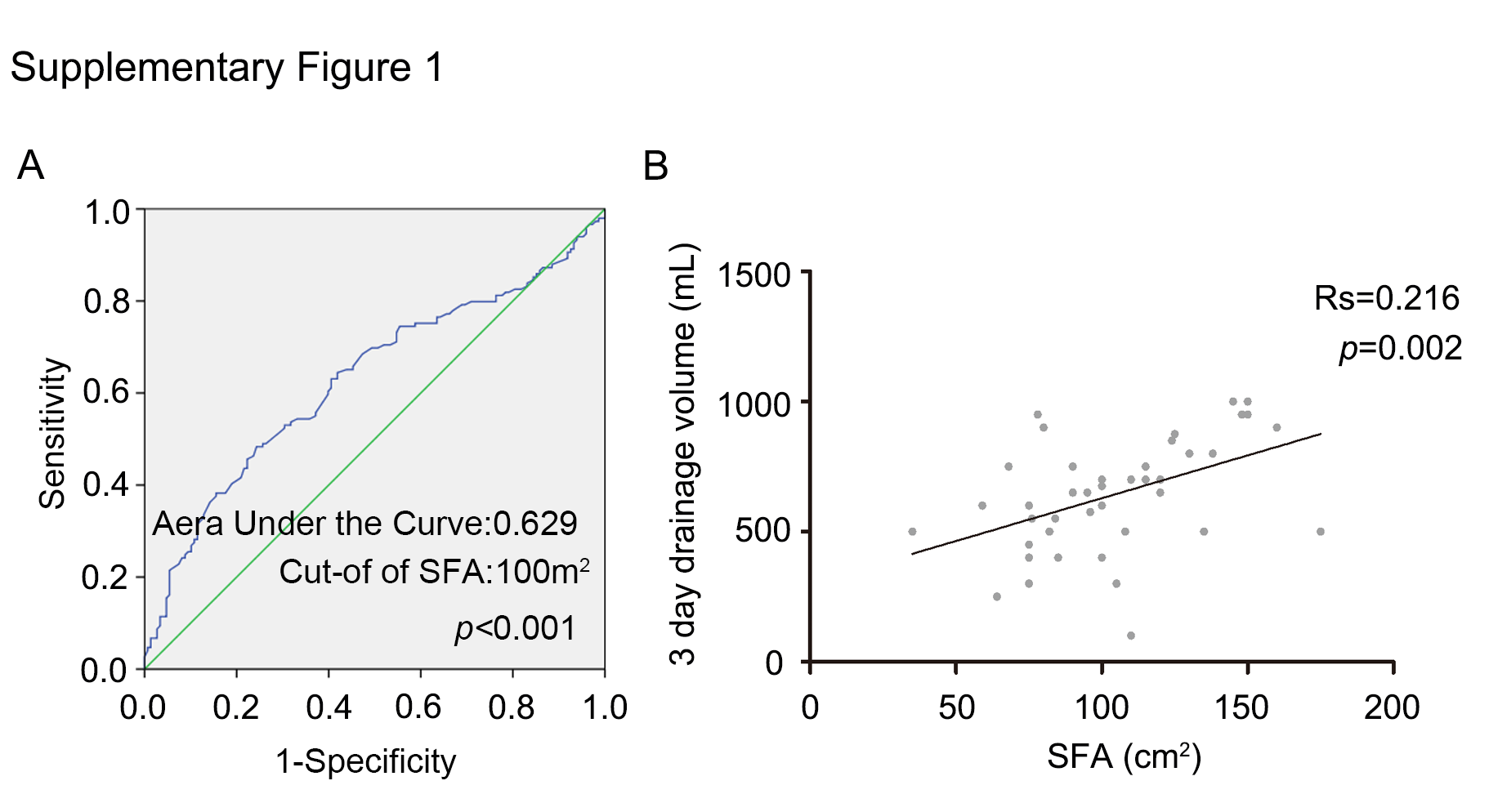

Supplement: Supplementary file 1 — Figure S1. Reactions between GPTMS and mesh. (A) ROC curve of SFA for 3‐day drainage volume (p < 0.001). (B) Correlation of SFA in 44 cases of new lung cancer patients undergoing lobectomy to 3‐day drainage volume (R s = 0.216, p = 0.002). [file TCA-16-e70114-s002.tif]
